# Implementation and Preliminary Evaluation of a Low-Cost Sodium Hypochlorite Shock Disinfection Protocol for Dental Unit Waterlines

**DOI:** 10.3390/dj14070451

**Published:** 2026-07-19

**Authors:** Letícia Silveira Carneiro, Maria Angélica Borba Vieira Ferreira, Lísia Aparecida Costa Gonçalves, Fabio Antonio Colombo, Amanda Latercia Tranches Dias, Marina Lara de Carli, Michelle Foigel Siqueira

**Affiliations:** 1 School of Dentistry, University of São Paulo, Ribeirão Preto 14040-904, SP, Brazil; leticia.carneiro@usp.br; 2 School of Pharmaceutical Sciences, Federal University of Alfenas, Alfenas 37130-001, MG, Brazil; maria.vieira@sou.unifal-mg.edu.br (M.A.B.V.F.); fabio.colombo@unifal-mg.edu.br (F.A.C.); 3 School of Dentistry, Federal University of Alfenas, Alfenas 37130-001, MG, Brazil; lisia.goncalves@unifal-mg.edu.br (L.A.C.G.); marina.carli@unifal-mg.edu.br (M.L.d.C.); 4 Institute of Biomedical Sciences, Federal University of Alfenas, Alfenas 37130-001, MG, Brazil; amanda.dias@unifal-mg.edu.br; 5 College of Dentistry, University of Saskatchewan, Saskatoon, SK S7N 5E4, Canada

**Keywords:** dental unit waterlines, decontamination, infection control, dental public health, sodium hypochlorite

## Abstract

**Background/Objectives:** This exploratory study aimed to characterize the microbiological contamination of dental unit waterlines (DUWLs) and to conduct a preliminary evaluation of a 0.5% sodium hypochlorite shock treatment. **Methods:** Water samples from five dental units in a single teaching institution were collected from the air/water syringe and high-speed handpiece lines at four time points: baseline, immediately after a shock treatment, and 7 and 14 days post-treatment. The water supply source was also analyzed. The intervention consisted of a 15-min exposure to 0.5% sodium hypochlorite. Bacterial and fungal contamination was assessed using validated semi-quantitative microbial samplers, and key bacterial isolates were identified. **Results:** Baseline assessments revealed substantial bacterial contamination in most units (≥301 CFU/mL), despite minimal contamination in the water supply source (<10 CFU/mL). An immediate reduction in microbial counts was observed following the shock treatment for both bacteria and fungi counts; however, resilient species such as *P. aeruginosa* and the spore-forming *B. cereus* persisted, while *K. pneumoniae* was no longer detected. Bacterial levels increased again within 7 days in several units, while fungal contamination remained low at the follow-up. At 14 days, bacterial results were variable and inconsistent. In contrast, fungal contamination remained suppressed in most units throughout the follow-up period. **Conclusions:** The sodium hypochlorite shock treatment produced an immediate decrease in microbial load in DUWLs, but the findings suggest that its sustained effect on bacterial control may be limited. Given the small sample size and single-center design, these results should be interpreted cautiously. The study demonstrates the feasibility of implementing a structured DUWL monitoring and maintenance program using a simple, low-cost sodium hypochlorite protocol, providing a practical model for improving water quality and patient safety in dental clinics.

## 1. Introduction

Infection control is a cornerstone of dental biosafety, aimed at preventing cross-infection from sources such as contaminated surfaces, instruments, air, and water [1,2]. A significant and often underestimated vector for microbial transmission is the dental unit waterline (DUWL), a network of narrow-bore plastic tubing that supplies water to dental instruments [1,3]. Water supplied to dental units may come from either open systems, which are directly connected to the municipal water supply, or closed systems, which use an independent reservoir. Regardless of the water source, DUWLs are highly susceptible to biofilm formation, posing potential health risks to both patients and dental professionals [4,5,6,7].

Biofilm development is multifactorial, stemming from microorganisms present in the source water and the retraction of patient saliva into the tubing, particularly in units lacking anti-reflux valves [2,5,6,8,9]. The inherent design of DUWLs creates an ideal environment for the proliferation of complex microbial communities, including bacteria, fungi, and protozoa. Among these, bacterial contamination is typically the most predominant [2,5,6,10,11,12,13].

DUWL biofilms frequently harbor high concentrations of opportunistic pathogens. Gram-negative environmental bacteria such as *Legionella*, *Pseudomonas*, and *Mycobacterium* species are of particular concern, as they can cause conditions ranging from mild inflammation to severe lung infections and septic shock [5,6,12,14,15,16]. Fungal contaminants, notably *Candida* spp., *Alternaria, Aspergillus,* and *Penicillium* are also present and can trigger allergies and hypersensitivity pneumonitis [17,18,19]. Despite these risks, fungal contamination remains largely unaddressed in official water quality regulations.

Chemical disinfection is the most recommended method for managing DUWL contamination [8,16,20,21]. However, the complete removal of established biofilms remains a challenge, in part due to microbial resistance, lack of standardized international regulations for DUWL cleaning and disinfection protocols, and insufficient education on DUWL maintenance during the training of oral healthcare professionals [3,14].

Although numerous studies have demonstrated the antimicrobial efficacy of sodium hypochlorite and other disinfectants against DUWL biofilms [8,16,20,21], translating these findings into standardized, sustainable maintenance protocols for routine clinical practice remains a challenge [8,22,23,24]. Many dental schools and private practices continue to lack structured DUWL monitoring and shock disinfection programs, highlighting the need for practical, evidence-based implementation strategies to improve water quality and patient safety.

In Brazil, one of the most commonly used disinfectants in hospital and dental settings is sodium hypochlorite. The use of 0.5% sodium hypochlorite for disinfecting DUWLs offers several practical and operational advantages, including broad-spectrum antimicrobial activity, low cost, widespread availability, and ease of implementation. These characteristics make it an attractive option for institutions seeking to establish sustainable and affordable DUWL maintenance programs, particularly where specialized commercial products may not be readily available [25,26,27].

To address this gap, this exploratory study aimed to evaluate the microbiological contamination of dental unit waterlines in a university dental clinic that had no previous routine monitoring or shock disinfection program and to implement and evaluate a simple, low-cost shock disinfection protocol using 0.5% sodium hypochlorite. Beyond assessing microbiological outcomes, the study sought to determine whether an accessible, evidence-based maintenance protocol could be successfully integrated into routine clinical practice to improve water quality and strengthen patient safety. By providing a practical implementation model, this work may assist other dental schools and clinical practices in establishing sustainable DUWL quality assurance programs.

## 2. Materials and Methods

### 2.1. Dental Unit Selection

This exploratory longitudinal implementation study evaluated microbial contamination in dental unit waterlines (DUWLs) before and after the introduction of a standardized shock disinfection protocol. Five dental units (Versa Max Plus, 2023; Dabi Atlante, São Paulo, Brazil) were randomly selected using Randomizer.org. All dental units within the clinic were identical with respect to design, operational lifespan, water supply, maintenance history, frequency of use, and clinical specialties served. Prior to the initiation of this study, the institution did not have a routine protocol for DUWL monitoring or scheduled shock disinfection. Consequently, five units were selected as a representative sample to evaluate the implementation of the proposed maintenance protocol. As this was an exploratory implementation study, no formal a priori sample size calculation was performed. Repeated microbiological assessments at four predefined time points were used to characterize changes following the intervention and generate preliminary data for future studies.

### 2.2. Sample Collection

Water samples were collected from the dental units at four time points: before the shock disinfection protocol (baseline), immediately after the shock disinfection protocol, and 7 and 14 days after the shock disinfection protocol. For each of the five dental units, a pooled water sample was prepared by collecting equal volumes of water from the air/water syringe and the high-speed handpiece into a single sterile container. The samples were pooled with the intention to simulate the combined microbial exposure encountered during routine dental procedures. Water samples were processed according to the manufacturer’s instructions using the HPC Total Count Sampler for bacterial analysis and the Yeast & Mold Sampler for fungal analysis (Millipore^®^, Sigma-Aldrich, Burlington, MA, USA). As controls, two samples were collected directly from the common filtered water supply supplying all five dental units, namely one for bacterial analysis and one for fungal analysis. Because all dental units received water from the same source, these samples were considered representative of the incoming water supplied to all units and therefore only one bacterial and one fungal control sample were collected.

### 2.3. Shock Disinfection Protocol

The shock disinfection protocol involved attaching an independent reservoir containing a 0.5% sodium hypochlorite solution to the dental unit. The disinfectant was flushed through the DUWLs until the tubing was fully filled, ensuring complete contact with all internal surfaces. The solution was then left in the lines for a 10–15-min contact period, consistent with manufacturer recommendations for bleach-based disinfection of dental systems. After the contact time, the disinfectant reservoir was removed and replaced with the unit’s filtered water bottle, and the lines were flushed for 2 min to eliminate any residual sodium hypochlorite before clinical use.

### 2.4. Microbiological Quantification and Bacterial Identification

Water samples were initially analyzed for semi-quantitative enumeration of bacterial and fungal contamination using the previously described HPC Total Count Sampler and Yeast & Mold Sampler (Millipore^®^, Sigma-Aldrich, Canada). The HPC Total Count Sampler was incubated at 35 °C for 7 days according to the manufacturer’s instructions. Although the manufacturer’s protocol for the Yeast & Mold Sampler recommends incubation at 28 °C for 72 h, preliminary testing conducted prior to this study demonstrated that fungal growth was consistently detectable only after incubation at 30 °C for 7 days. Therefore, these incubation conditions were adopted throughout the study. Two independent researchers quantified microbial growth by assigning the corresponding colony-forming unit (CFU/mL) category according to the manufacturer’s interpretation chart, similar to the methodology described by Buitrago, Kolbe and Siqueira [28].

Bacterial identification was subsequently performed only for samples collected immediately before and immediately after the shock disinfection protocol. Samples obtained at 7 and 14 days were used solely for semi-quantitative microbial enumeration and were not subjected to species-level bacterial identification.

For bacterial identification, samples were cultured on Brain Heart Infusion (BHI) agar and incubated at 35 °C for 48 h. After incubation, microorganisms were examined based on colony morphology and Gram staining profiles. Initial identification of bacterial isolates was based on colony morphology and Gram stain reactions, followed by complementary biochemical testing performed according to standard microbiological procedures to support phenotypic identification. Among the biochemical tests performed for Gram-negative bacteria, oxidase activity was evaluated to determine the presence of cytochrome c oxidase, a key enzyme of the aerobic respiratory chain. This test was used to differentiate major bacterial groups, particularly members of the *Enterobacteriaceae* family (oxidase-negative) from non-fermenting Gram-negative bacilli (oxidase-positive). Additional biochemical and phenotypic characteristics evaluated included growth at 42 °C, production of pyoverdine and pyocyanin, lactose fermentation, lysine decarboxylase activity, motility, and indole production. For Gram-positive bacteria, catalase activity, oxidase activity, and motility were assessed. Reference strains were not included because the objective was routine phenotypic characterization of environmental isolates rather than diagnostic validation or molecular confirmation.

### 2.5. Statistical Analysis

Data were analyzed using descriptive statistical methods in Microsoft Excel 365. Given the exploratory implementation design, the repeated-measures nature of the data, and the limited sample size, analyses were restricted to descriptive summary measures. Microbial counts (CFU/mL) were categorized according to predefined thresholds and summarized across time points. Categorical variables were reported as frequencies and proportions, with results presented in tables and figures to facilitate comparison across sampling intervals. No inferential statistical analyses were performed because the study was not designed or powered for hypothesis testing.

### 2.6. Ethics

A letter of exception was obtained prior to the beginning of this study. Ethical approval was not required for this study.

## 3. Results

### 3.1. Baseline Contamination and Water Source Analysis

**Figure 1:**
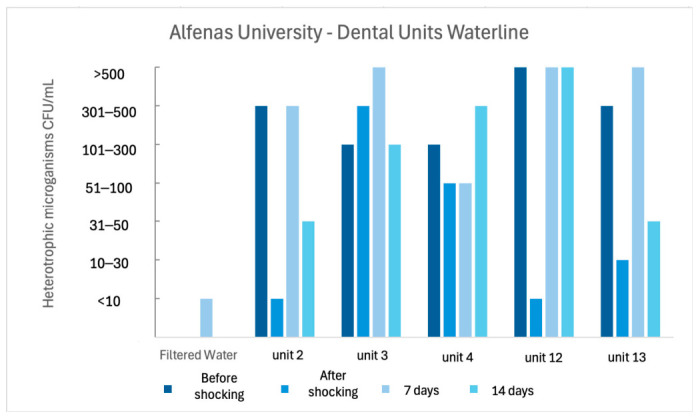
Bacterial contamination levels in the DUWLs of five dental units were evaluated at baseline, immediately after the shock disinfection protocol, and again at 7 and 14 days post shock disinfection protocol.

**Figure 2:**
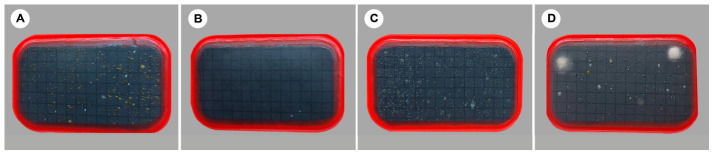
Bacteria counts at (**A**) baseline, (**B**) immediately following shock disinfection protocol, (**C**) at 7-day follow-up, and (**D**) at 14-day follow-up of unit 2. The images were chosen based on typical colony morphology and CFU density within each categorical range.

**Figure 3:**
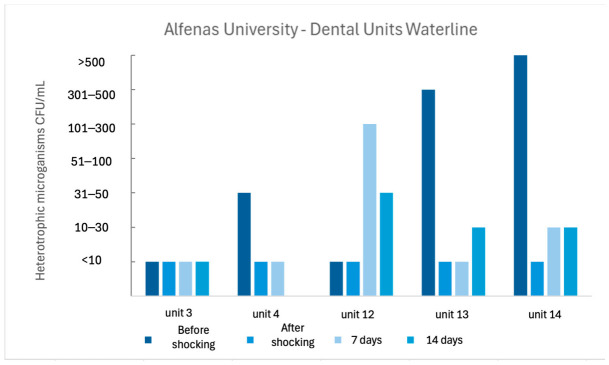
Fungal contamination levels in the waterlines of five dental units were quantified at baseline, immediately following shock disinfection protocol, and at 7- and 14-day post shock disinfection protocol intervals.

The filtered municipal water supply showed minimal contamination (<10 CFU/mL for both bacteria and fungi). In contrast, all five DUWLs exhibited bacterial loads >100 CFU/mL at baseline, prior to any intervention, with considerable variability among units (Table 1, Figure 1 and Figure 2A). Units 2, 12, and 13 presented the highest bacterial categories (301–500 or >500 CFU/mL), whereas units 3 and 4 showed intermediate levels (101–300 CFU/mL). Initial fungal contamination was considerably lower than bacterial levels, with most of the units (60%) registering counts at or below 50 CFU/mL (Figure 3). Baseline fungal levels varied significantly across the units: units 3 and 12 showed minimal fungal presence (<10 CFU/mL); unit 4 exhibited slightly higher baseline categories (31–50 CFU/mL); and units 13 and 14 presented high contamination (301–500 CFU/mL and >500 CFU/mL, respectively). Species-level bacterial identification was performed only for samples collected immediately before and immediately after the shock disinfection protocol. Samples collected at 7 and 14 days were analyzed only for quantitative CFU/mL enumeration. (Table 1).

Baseline bacterial identification confirmed polymicrobial contamination across the DUWLs, with isolates including *Pseudomonas aeruginosa*, *Klebsiella pneumoniae*, and *Bacillus cereus* (Table 1). Among these, *Pseudomonas aeruginosa* and *Klebsiella pneumoniae* were the most frequently detected species, each present in four of the five units before treatment. At baseline, bacterial contamination was high in all units, with three units (60%) presenting levels ≥301 CFU/mL and two units (40%) showing levels between 100–300 CFU/mL.

### 3.2. Efficacy of Shock Disinfection Protocol on Bacteria

The 0.5% sodium hypochlorite shock disinfection protocol produced an immediate reduction in bacterial contamination in four of the five dental units (80%). Units 2 and 12 showed a marked reduction from ≥301 CFU/mL to <10 CFU/mL, while Unit 13 decreased from 301–500 CFU/mL to 10–30 CFU/mL. Unit 4 exhibited a more modest reduction, from 101–300 CFU/mL to 51–100 CFU/mL. In contrast, Unit 3 (20%) showed an increase in bacterial contamination from 101–300 CFU/mL to 301–500 CFU/mL immediately after the shock disinfection protocol (Table 1; Figure 1 and Figure 2B). Microbial identification revealed that *Pseudomonas aeruginosa* and *Bacillus cereus* remained detectable in units with persistent bacterial contamination, whereas *Klebsiella pneumoniae* was no longer identified after treatment (Table 1). In Units 2, 12, and 13, bacterial identification following the shock disinfection protocol was recorded as “unidentified” because bacterial growth was absent or insufficient to support reliable phenotypic identification using the culture and biochemical methods employed. Furthermore, two distinct Gram-negative bacillary profiles were observed: one corresponding to *P. aeruginosa*, characterized by positive oxidase activity, growth at 42 °C, and production of pyoverdine and pyocyanin, and the other corresponding to *K. pneumoniae*, which exhibited negative oxidase activity, lactose fermentation, lysine decarboxylase positivity, motility, and negative indole production. A Gram-positive bacillus consistent with *B. cereus* was also identified, showing positive catalase, positive oxidase activity, and motility.

### 3.3. Bacterial Recolonization at 7 and 14 Days

The suppressive effect of the 0.5% sodium hypochlorite shock treatment was temporary, leading to highly unit-specific recolonization patterns. At day 7, four units (2, 3, 12, and 13) rebounded to high contamination levels (≥301 CFU/mL or >500 CFU/mL), whereas unit 4 remained stable at 51–100 CFU/mL (Figure 1). By day 14, variability increased further: unit 12 sustained maximum contamination (>500 CFU/mL), while unit 4 experienced a marked increase, rising to 301–500 CFU/mL. In contrast, unit 3 decreased to 101–300 CFU/mL, and units 2 (Figure 2D) and 13 showed substantial reductions, dropping to the 31–50 CFU/mL category (Figure 1).

### 3.4. Efficacy of Shock Disinfection Protocol on Fungi

The 0.5% sodium hypochlorite shock protocol produced an immediate reduction in fungal load, with units 13 and 14 showing a pronounced shift from high contamination to <10 CFU/mL, and unit 4 showed a reduction from 31–50 CFU/mL to <10 CFU/mL. Units 3 and 12 maintained their initial minimal fungal presence (<10 CFU/mL) immediately after treatment.

### 3.5. Fungal Recolonization at 7 and 14 Days

**Figure 4:**
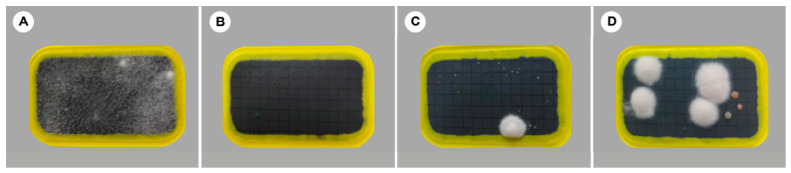
Fungi counts at (**A**) baseline, (**B**) immediately following shock disinfection protocol, (**C**) at 7-day follow-up, and (**D**) at 14-day follow-up of unit 14. The images were chosen based on typical colony morphology and CFU density within each categorical range.

The suppressive effect was not uniformly maintained throughout the follow-up period. Units 3, 4, and 13 maintained minimal fungal levels (<10 CFU/mL) up to day 7; however, by day 14, unit 13 showed a slight increase to the 10–30 CFU/mL category. Only units 3 and 4 remained at <10 CFU/mL throughout the entire 14-day period. Unit 14 exhibited early recolonization, reaching 10–30 CFU/mL at day 7 and maintaining this level at day 14 (Figure 4). Additionally, unit 12 experienced a notable spike in fungal contamination at day 7 (101–300 CFU/mL), which subsequently decreased to 31–50 CFU/mL by day 14. Consequently, patterns of fungal recolonization were highly unit-specific and occurred in multiple units rather than being limited to a single one (Figure 3 and Figure 4C,D).

## 4. Discussion

The initial results indicate a concerning level of bacterial and fungal contamination in DUWLs that were not subjected to a routine shock disinfection protocol. Regarding best practices in dental healthcare settings, different jurisdictions have different rules regarding the number of bacteria that can be present in water from dental water units [6]. The heterotrophic bacterial count should not exceed 500 CFU/mL according to both the Brazilian national drinking water standard and the U.S. Centers for Disease Control and Prevention (CDC) [7,28]. In this study, 20% of samples exceeded this limit, and an additional 60% fell within 301–500 CFU/mL. These findings are consistent with previous research reporting that 21% of DUWL samples surpassed 500 CFU/mL [28], as well as studies documenting levels above the American Dental Association (ADA) recommendation of <200 CFU/mL [5,9,12,14]. Additionally, 20% of samples demonstrated fungal contamination; however, no national or international standards have been established for acceptable fungal levels in dental unit water [21].

Despite the current recommendation to supply dental units with distilled or sterilized water, or directly from public water supplies rather than reservoirs [6,8,12,20], the present study found no significant contamination in the source water, indicating that the filtered municipal water supply is suitable for use. This finding suggests that the biofilm formed within the DUWLs originates from stagnant water and/or saliva backflow into the tubing system during dental procedures. A systematic review conducted by Wu et al. [25] highlighted the recommendation of using independent water reservoirs for disinfecting DUWLs with distilled water, as well as the need to combine flushing DUWLs with disinfection.

Application of the 0.5% sodium hypochlorite shock protocol resulted in a reduction in bacterial CFU/mL in 80% of the units immediately after the shock treatment. However, one unit showed an increase in microbial counts. Although this study cannot determine the underlying cause, one possible hypothesis is that sodium hypochlorite may have dislodged portions of the existing biofilm, releasing previously embedded microorganisms into the water [23]. Contamination levels may also be influenced by the types of dental procedures performed in each unit, inconsistent or inadequate infection control practices, including failure to purge waterlines at the beginning of the day and between appointments, and differences in microbial composition [29,30]. These findings underscore the importance of routine monitoring of water quality and biofilm characterization in DUWLs, given the likelihood of detecting opportunistic pathogens [7,31].

Ideally, a decontamination agent for dental settings should be cost-effective, biocompatible, and have minimal impact on the structural integrity of DUWL components [6,10,20,21,32]. Sodium hypochlorite remains the most widely employed disinfectant in dental environments [4,8,10,15,19,20,29,30,32,33], although alternative agents such as chlorine dioxide [10,11] and hypochlorous acid [11,18,19,34,35] have also been reported. However, the performance of chlorine-based disinfection protocols can be inconsistent due to the absence of standardized procedures [3,36]. To properly disinfect DUWL biofilms, it is advised to adopt a comprehensive approach that includes physical, chemical, and automated approaches in accordance with the normal legal compliance standards [6].

Although prolonged use of sodium hypochlorite can damage waterlines through electrochemical reactions, this effect can be minimized by using lower concentrations. Effective microbial reduction has been reported at concentrations as low as 0.1% [20]. In the present study, the application of 0.5% sodium hypochlorite for 10–15 min, followed by immediate flushing with filtered water, did not result in any observable deterioration of the tubing system, supporting both its efficacy and practicality.

Microbial identification revealed the presence of clinically relevant opportunistic pathogens within the DUWLs, including *Pseudomonas aeruginosa*, *Klebsiella pneumoniae*, and *Bacillus cereus*. The detection of *P. aeruginosa* is particularly notable, as this Gram-negative bacillus is a well-recognized biofilm former frequently associated with DUWL contamination and healthcare-associated infections [12]. The presence of *K. pneumoniae* and the spore-forming *B. cereus* further highlights the potential risk posed to both patients and dental personnel. In the same direction, a systematic review revealed a significant incidence of bacterial biofilm in DUWLs; as a result, using the proper disinfectants is advised to lessen the risk of infection and the prevalence of contamination [5].

Post-treatment analysis demonstrated variable responses to sodium hypochlorite. While *K. pneumoniae* was successfully eliminated, *P. aeruginosa* and *B. cereus* persisted in two units, indicating greater tolerance to the disinfectant. The anomalous increase in microbial load observed in Unit B, where both *B. cereus* and *P. aeruginosa* reappeared, supports the hypothesis that shock treatment may dislodge existing biofilm deposits rather than achieve complete eradication [23]. The resilience of *B. cereus* is especially concerning, as its endospores exhibit high resistance to chemical disinfectants and can facilitate rapid recolonization [37,38]; additionally, Lineback et al. [33] observed that products containing hydrogen peroxide and sodium hypochlorite are effective against biofilms of *S. aureus* and *P. aeruginosa*, and that bactericidal activity against biofilms generally varied depending on the active ingredient.

These findings highlight that a single disinfection protocol may not be uniformly effective against the diverse microbial communities present in DUWLs and underscore the need for strategies capable of addressing persistent and spore-forming species. Chlorine-based disinfectants are commonly employed to manage microbiological contamination in DUWL [11]; however, brief contact decreases bacterial colonization without eliminating the biofilm. When comparing the disinfection of water lines in dental offices with the disinfection of root canals, 1% NaOCl and 2% CHX have little antibiofilm activity, whereas NaOCl (at 3% and 6%) effectively destroys bacteria and damages the extracellular matrix of mature biofilms composed of various species [39].

The 7-day post-treatment assessment revealed substantial bacterial recolonization in all units, with 60% exhibiting counts above 500 CFU/mL. Although this study was not designed to identify the mechanisms driving these fluctuations, the results clearly demonstrate that a single shock treatment did not prevent subsequent recolonization. In addition, bacterial isolates from Units 2, 12, and 13 could not be reliably identified immediately after the shock disinfection protocol because bacterial growth was absent or insufficient to support phenotypic identification using the microbiological methods employed. These findings likely reflect a marked reduction in bacterial burden following treatment rather than complete microbial elimination. Re-contamination after disinfection has been reported in the literature, particularly when regular monitoring and maintenance protocols are lacking [8,11]. In contrast, only one unit showed notable fungal recolonization, suggesting that fungal growth in DUWLs may occur more slowly than bacterial regrowth. Another hypothesis is that the rapid and dense bacterial proliferation observed at 7 days could have limited fungal growth; however, this study was not designed to evaluate competitive interactions, and this explanation should be interpreted only as a possible mechanism. Besides that, concerns have been raised regarding the gradual release of potentially dangerous materials from the remaining biofilm matrix, due to the long-term effects of these agents. Even with periodic treatments, these compounds reduce the number of bacteria in wastewater but have little effect on the DUWL biofilm matrix [40].

The 14-day post-shock evaluation demonstrated variable bacterial outcomes, with 40% of units showing a reduction in contamination relative to the 7-day assessment. This fluctuation suggests that recolonization of dental unit waterlines is a dynamic and non-linear process rather than a continuous increase in microbial burden. This variability could reflect differences in clinical procedures performed across units; however, this should be interpreted only as a hypothesis, as the study was not designed to evaluate the influence of clinical disciplines or patterns of waterline use on recolonization. These environments differ in frequency and volume of waterline use, a factor known to influence microbial accumulation. Regular and continuous water flow can reduce biofilm formation, whereas sporadic use may facilitate its growth. Regarding fungal activity, an 80% reduction in CFU was observed at 14 days compared to pre-shock levels. Despite the efficacy of the disinfectant, previous studies have reported increases in fungal colony size or fungal contamination following treatment [29], demonstrating variability in fungal responses. Future studies should examine how specific clinical procedures correlate with DUWL water quality.

There is currently no consensus on the optimal timing or frequency of DUWL decontamination, whether before first clinical use, daily prior to patient care, between appointments, or as part of a combined regimen using low-concentration daily treatments supplemented by periodic high-concentration shock protocols [15,41]. In the present study, the protocol was performed once at the beginning of the clinical session. Continuous disinfection reduces bacterial levels in DUWLs more effectively than intermittent disinfection [25]. The likelihood of the formation of microbiological strains resistant to disinfectants can be decreased by routinely rotating disinfectants.

This study evaluated bacterial and fungal contamination using the HPC Total Count Sampler and Yeast & Mold Sampler (Millipore^®^, Sigma-Aldrich, Canada). Although the manufacturer’s protocol for the Yeast & Mold Sampler recommends incubation at 28 °C for 72 h, preliminary testing conducted prior to this study demonstrated that fungal growth was consistently detectable only after incubation at 30 °C for 7 days. Therefore, these incubation conditions were adopted throughout the study to optimize the recovery of environmental fungi from DUWL samples. The HPC Total Count Sampler and Yeast & Mold Sampler provide practical, reproducible, and standardized methods for routine DUWL monitoring [11,28]. The conventional surface swab method has also been reported for assessing these microorganisms [8,29]; however, the Sigma-Aldrich^®^ waterline test kits offer greater ease of use and can be readily implemented in the routine of dental practices.

Beyond the microbiological findings, an important contribution of this study is the demonstration that a simple, low-cost DUWL maintenance protocol can be successfully implemented in a university dental clinic. At the time this study was initiated, the institution did not have a structured protocol for routine monitoring or shock disinfection of dental unit waterlines. The implementation of an evidence-based maintenance strategy using readily available 0.5% sodium hypochlorite established a sustainable quality assurance approach that can be integrated into routine clinical practice. Although additional studies are needed to determine the optimal frequency of monitoring and shock disinfection, this implementation model demonstrates that meaningful improvements in water quality and patient safety can be achieved using readily available resources. Given that many dental schools and private practices continue to lack standardized DUWL maintenance programs, the present findings may provide a practical framework for the development of similar quality assurance initiatives.

Although the microbiological results were promising, several limitations should be acknowledged. Given the exploratory nature of our study, the small sample size (five dental units), the relatively short 14-day follow-up period, and the single-center design, the application of inferential tests would not yield statistically meaningful or generalizable results. In such conditions, inferential statistics would risk producing misleading interpretations due to insufficient statistical power. For these reasons, descriptive statistics are the most appropriate and scientifically sound approach for accurately reporting the microbiological behavior observed in each unit over time. The use of composite samples from the air/water syringe and high-speed handpiece prevented independent evaluation of each waterline; however, in the context of this exploratory study, pooling the water from the air/water syringe and high-speed handpiece of the same dental unit was intentionally performed to simulate their concomitant clinical use during dental procedures. The objective was to evaluate the combined biofilm released from both waterlines, which reflects the real exposure scenario for patients and practitioners. An additional limitation is that microbiological sampling was performed immediately after treatment and at 7 and 14 days, without intermediate evaluations. Consequently, the exact timing of microbial recolonization could not be determined. Future longitudinal studies should include earlier post-treatment sampling (e.g., 2–3 days) to better characterize the kinetics of bacterial and fungal recolonization following shock disinfection.

While the HPC Total Count Sampler and Yeast & Mold Sampler used in this study provide semi-quantitative categorical ranges rather than precise colony-forming unit (CFU) counts, they represent validated and widely used methods for routine DUWL monitoring because of their practicality, reproducibility, and suitability for clinical settings. In addition, this exploratory study was conducted in a single university clinic using a limited number of randomly selected dental units. Consequently, the findings should be interpreted as preliminary and may not be generalizable to all clinical settings. Nevertheless, they provide a foundation for future multicenter studies evaluating practical DUWL maintenance strategies in routine clinical practice. Finally, the lack of standardized international recommendations regarding disinfectants, concentrations, contact times, monitoring intervals, and maintenance schedules continues to limit comparisons across studies and highlights the need for multicenter investigations to establish evidence-based guidelines for routine DUWL quality assurance.

## 5. Conclusions

This exploratory study identified a high prevalence of bacterial and fungal contamination in DUWLs that were not managed under a structured DUWL maintenance program incorporating routine shock disinfection, with microbial loads frequently exceeding recommended safety thresholds. The implementation of a simple, low-cost shock disinfection protocol using 0.5% sodium hypochlorite resulted in an immediate reduction in microbial contamination without observable damage to the dental unit waterlines, demonstrating that an accessible and readily available disinfectant can be effectively incorporated into routine clinical practice.

Although bacterial recolonization was observed within seven days, indicating that a single shock treatment is insufficient to provide sustained microbial control, the findings reinforce the importance of establishing routine monitoring and structured maintenance programs rather than relying on sporadic interventions. Importantly, this study demonstrates that a structured, evidence-based DUWL maintenance program can be successfully implemented in a university dental clinic that previously had no routine monitoring or shock disinfection protocol.

Future multicenter studies with larger sample sizes and longer follow-up periods are warranted to establish evidence-based recommendations regarding the optimal frequency of monitoring and shock disinfection for long-term DUWL quality assurance. Ultimately, improving dental unit water quality depends not only on selecting an effective disinfectant, but also on implementing sustainable monitoring and evidence-based maintenance protocols as integral components of routine clinical practice.

## Figures and Tables

**Table 1:** Level of bacterial contamination according to the dental unit collected immediately before and after shock disinfection protocol.

DUWL	Water Collection Period and Type of Microorganism Assessed			
	Before Shocking	After Shocking	Before Shocking	After Shocking
	CFU/mL Bacteria		Bacterial Identification	
2	301–500	<10	*Klesiella pneumoniae* and *Pseudomonas aeruginosa*	unidentified
3	101–300	301–500	*Bacillus cereus* and *Pseudomonas aeruginosa*	*Bacillus cereus* and *Pseudomonas aeruginosa*
4	101–300	51–100	*Bacillus cereus, Klebsiella pneumoniae* and *Pseudomonas aeruginosa*	*Bacillus cereus*, *Pseudomonas aeruginosa*
12	>500	<10	*Kllebsiella pneumoniae* and *Pseudomonas aeruginosa*	unidentified
13	301–500	10–30	*Klebsiella pneumoniae* and *Pseudomonas aeruginosa*	unidentified

## Data Availability

All data generated or analyzed during this study are included in this published article.

## Abbreviations

The following abbreviations are used in this manuscript:

DUWL	Dental unit waterline
CFU	Colony-forming units per milliliter
BHI	Brain Heart Infusion
ADA	American Dental Association
